# Natural Autoantibodies: An Undersugn Hero of the Immune System and Autoimmune Disorders—A Point of View

**DOI:** 10.3389/fimmu.2018.01320

**Published:** 2018-06-12

**Authors:** Stratis Avrameas, Haris Alexopoulos, Harry M. Moutsopoulos

**Affiliations:** ^1^Laboratory of Immunology, Hellenic Pasteur Institute, Athens, Greece; ^2^Department of Pathophysiology, Faculty of Medicine, National and Kapodistrian University of Athens, Athens, Greece; ^3^Academy of Athens, Athens, Greece

**Keywords:** autoantibodies, autoimmunity, immune system, natural autoantibodies, polyreactivity

Natural autoantibodies/natural autoimmunity forms a network that serves to protect the organism from outer and inner danger but may also contribute to autoimmune disease. This vital component of the immune system deserves, we think, much greater attention than it currently attracts. A better understanding of how natural autoimmunity contributes in the function of the immune system is needed and how the expansion of this process leads to autoimmune disorders is an intriguing question which we will address in this article.

Since the early days of immunology, pioneering work by Landsteiner and his group on haptens showed that immunization is a process that can raise highly specific antibodies. In the same era, Ehrlich put forward the *dogma* of *“horror autotoxicus,”* proclaiming the inability of the immune system to recognize self-constituents. Later on, in the 1950s, Burnet builds upon these seminal observations and formulated the clonal selection theory, which states that *“cell clones produce highly specific antibodies for environmental constituents while the immune system is educated during ontogeny not to recognize self-constituents.”*

Nevertheless, other studies, contradicting the “dogma,” were mostly ignored. These studies showed that circulating antibodies in normal human sera could recognize various substances, including self-antigens, albeit with a low avidity (1, 2). In the following years, additional experiments reinforced the notion of a persistent immune system reactivity against self, e.g., in normal mice (3, 4).

Many of these studies were neglected because the scientific community had adopted the “dogma” of clonal selection theory. Despite this opposition, “heretical” studies kept coming and demonstrated that autoreactive B-cells and polyreactive antibodies are abundant and actively participate in physiological immunity (5). In mice, innate-like B1 cells form a pool of long-lived population, which are positively selected for self reactivity and produce the majority of natural IgM antibodies. These antibodies protect against infection and are implicated in the gut microbiota homeostasis (6).

## The Role of Polyreactivity in Natural Defenses

One of the main and consistent findings of these “heretical” studies we mentioned was that despite the undisputed distinction in the functions of B and T cells, both express a common feature that appears to be preserved during the evolutionary process; auto-polyreactivity (7, 8). In invertebrates and lower vertebrates, recognition of self-antigens is suggestive of an evolutionary process where multicellular organisms, probably in order to protect themselves from ominous invaders, began to produce both B- and T-cell receptors recognizing environmental constituents, derived from proteins employed in their embryonic development. In order to be able to recognize environmental constituents, they had to lose part of their ability to recognize self. In the course of evolution, these auto-polyreactive immune receptors ended up recognizing more avidly the external rather than the internal constituents of the organism (9). Higher vertebrates maintained the ability to produce these auto-polyreactive immune receptors. Since then, vertebrates acquired the additional capacity to produce specific receptors that recognize environmental antigens, yet, their ability to recognize self-antigens, *via* polyreactivity was maintained; an evolutionary genetic fossil maintained in the germline (10, 11). Is this ancient aspect of the immune system still relevant for day-to-day immunity? This network of low-specificity polyreactive natural antibodies is still crucial for instantly recognizing an acute infection and marking the invader for the first responders of the innate immunity. Their ability to bind both self and non-self molecules allows the binding of, e.g., cytokines and other self constituents, masking them from the adaptive immune system and, therefore, actively inhibiting pathogenic autoimmunity (12).

Under physiological conditions, auto-polyreactive immune receptors have their active site blocked by the high amount of available self-antigens. Auto-polyreactive immune receptors upon binding to self-antigens form immune complexes, which are subsequently eliminated from the circulation, primarily by phagocytosis (13). Auto-polyreactivity endows the immune system with the readily available capacity to recognize and interact with self and environment constituents, e.g., the gut microbiota, reminiscent of our ancestors (14). A vast network is created, which is changing perpetually depending upon the stimuli derived either from the internal or the external milieu of the organism contributing significantly to immune homeostasis. In disease states, where self-antigens are released, this network is a guardian that rapidly sequesters the self-antigens, e.g., in pathologic conditions such as demyelination, stroke, or pulmonary disease (15–17). Also, vital homeostatic biological activities such as cellular repairing and enzymatic catalysis are also monitored by several auto-polyreactive immune receptors (7, 18). These data clearly demonstrate that reactivity against self exists and that it has a well-defined physiological role.

## But What about Autoimmune Diseases?

The cardinal feature of an autoimmune disease is the unfolding of an excessive self-reactive, antigen-driven, immune response. The emergence of these disorders is considered a multifactorial process. Genetic predisposition (19, 20), environmental insults such as infections, chemical and physical agents (21), as well as stressful life events (22) all have been implicated. This self-addressed intense immune response is mediated by the physiological cytokine and immune cells repertoire and leads to organ dysfunction, hyperfunction, or tissue injury (23, 24). Are B- or T-cells that express natural autoantibody receptors the key links between natural self-polyreactivity and pathologic autoimmunity? To put it in other words, is there any evidence that excessive polyreactivity (including self-reactivity) can lead to disease? We propose that chronic activation of the immune system (25, 26) can lead to expansion of naturally present auto-polyreactive clones and, in genetically predisposed individuals, this can lead to the development of autoimmune disease. Our proposal is supported from clinical observations and experimental data.

It is well known that in sympathetic ophthalmia, release of hidden autoantigens in abundance, from, e.g., trauma of one eye are readily recognized by circulating preexisting autoreactive lymphocytic clones leading to autoimmune insult in the other eye (27). It is also well understood that chronic activation of the immune system, as occurs in chronic infections like subacute bacterial endocarditis (28), leishmaniosis (29), and others, may lead, in genetically susceptible individuals, to immune complex formation and development of autoimmune disease. Furthermore, many years ago, we have shown, followed by others that chronic activation of the immune system, as occurs in individuals who received allogeneic bone transplants and develop chronic graft versus host disease also leads to the development of a variety of systemic autoimmune diseases such as systemic lupus erythematosus (SLE), Sjögren’s syndrome, biliary cirrhosis, and scleroderma (30–32).

Another important clue comes from the newly described age-associated B cells, which progressively accumulate with age. The appearance of these cells is correlated with disease onset in several murine SLE models (33). These cells are driven by a T-bet centered transcriptional program and progressively accumulate throughout life. These cells may well represent polyreactive memory B-cells, which recognize chronic pathogenic microbes but also autoantigens. The relative abundance of these cells in aging individuals may be an explanation of why autoimmune reactivity is more common in the elderly (34). This notion has also been demonstrated previously, in studies where, in healthy elderly individuals, a high incidence of autoantibodies was observed, particularly of the IgG isotype (35, 36). It would be worth investigating further the spectrum of autoantibodies in the elderly and whether these are produced by the age-associated B cells.

Further proof is arising from studying pathogenetic aspects of Sjögren’s syndrome, an autoimmune epithelitis (37). It is known, from studies in our and other laboratories, that the key player who initiates and perpetuates the autoimmune reactivity is the activated epithelium of the affected organs. We, and others, have shown that in patients with Sjögren’s syndrome, aggressive lymphocytic infiltrates surround the epithelia of the affected organs such as the salivary glands, the bronchi, the renal tubules, and the cholangia. Epithelia from labial minor salivary glands remain activated, even in long-term cultures, since they retain inappropriate expression of immune-regulatory molecules such as B7, ICAM, CD40, lympho-attractant chemokines, and pro-inflammatory cytokines, die from apoptotic death and release exosomes. Both apoptotic blebs and exosomes contain abundance of the autoantigens Ro and La, which in tandem with the activated epithelium, chronically activate the immune system rendering it aggressively autoreactive. As a result, the already present natural autoreactive lymphocytic clones may expand leading to the destruction of the affected organ and the development of autoimmune epithelitis. The question why the salivary gland epithelium of patients with Sjögren’s syndrome is activated is a fundamental and intriguing question. Our studies have shown that coxsackie viral sequences are present in the salivary gland epithelium of patients with Sjögren’s syndrome. Further studies are needed, however, to explore the exact etiopathogenetic role of this finding. All the aforementioned findings are reviewed in detail (38).

## Conclusion

High avidity recognition, lack of specificity, and tolerance to self has been the dominant idea of the clonal selection theory that guided the thinking in immunology and autoimmunity. Nevertheless, results from both older and newer studies have established that the immune system can recognize self, principally through auto-polyreactive receptors. Only a minor part of the immune system expresses high specificity, while its largest part exhibits polyspecificity or otherwise polyreactivity. In genetically prone individuals, chronic immune activation may lead to expansion of autoreactive lymphocytic clones that can induce cell or organ damage and thus development of autoimmune disorders.

## Author Contributions

SA, HA, and HM: drafting, editing, and final approval of manuscript.

## Conflict of Interest Statement

The authors declare that the research was conducted in the absence of any commercial or financial relationships that could be construed as a potential conflict of interest. The reviewer JW and the handling Editor declared their shared University affiliation although working in different departments.
